# Supplementary material for: A Mallows-like criterion for anomaly detection with random forest implementation
Source: PLoS One. 2025 Jun 6;20(6):e0323333. doi: 10.1371/journal.pone.0323333 (PMC12143530; doi:10.1371/journal.pone.0323333)
Supplement: S1 Table — (PDF) [file pone.0323333.s001.pdf]

**Table 1.** Hyperparameters of different datasets.

| Dataset | Hyperparameters | | |
|---|---|---|---|
| | $\alpha$ | $\beta$ | w |
| SB | 1.6220 | 0.6626 | 0.0392 |
| Pis | 1.7169 | 0.5579 | 0.0445 |
| MHR | 1.7491 | 0.9754 | 0.0393 |
| PS | 2.6335 | 0.9972 | 0.0436 |
| PCO | 2.9073 | 0.9736 | 0.0434 |
| Ye | 1.1717 | 0.5579 | 0.0445 |
| Ca | 1.7491 | 0.9754 | 0.0393 |
| MF | 1.3920 | 0.5526 | 0.0230 |
| SA | 2.2207 | 0.6107 | 0.0490 |
