# Supplementary material for: A Mallows-like criterion for anomaly detection with random forest implementation
Source: PLoS One. 2025 Jun 6;20(6):e0323333. doi: 10.1371/journal.pone.0323333 (PMC12143530; doi:10.1371/journal.pone.0323333)
Supplement: S2 Table — (PDF) [file pone.0323333.s002.pdf]

Table 2. AUC scores of anomaly detection algorithms

Model \ Dataset	SB	Pis	MHR	PS	PCO	Ye	Ca	MF	Sa	Mean
Modified Focal	**0.8702**	**0.7873**	**0.5742**	0.7944	**0.8956**	**0.5789**	**0.7500**	**0.9656**	**0.9529**	**0.7966**
Focal	0.7231	0.5770	0.4951	0.7040	0.8141	0.4964	0.6500	0.9589	0.9134	0.7036
Vote	0.7121	0.5694	0.4959	0.7003	0.8118	0.4970	0.6167	0.9560	0.9110	0.6967
Zero One	0.7231	0.5770	0.4951	0.7040	0.8141	0.4964	0.6500	0.9589	0.9134	0.7036
Hamming	0.7231	0.5770	0.4951	0.7040	0.8141	0.4964	0.6500	0.9589	0.9134	0.7036
Hinge Loss	0.7355	0.6201	0.5323	0.7119	0.8032	0.5396	**0.7500**	0.9498	0.9030	0.7273
Cross Entropy	0.7335	0.6072	0.5388	0.7118	0.8080	0.5383	**0.7500**	0.9539	0.9064	0.7275
Average	0.7231	0.5185	0.4950	0.7040	0.8141	0.4964	0.6500	0.9589	0.9134	0.7036
IF	0.5071	0.5185	0.4604	0.7315	0.7975	0.4400	0.6250	0.4630	0.4232	0.5518
Logistic	0.6914	0.5000	0.5000	0.5000	0.5000	0.5000	0.5000	0.5000	0.5000	0.5213
KNN	0.5185	0.5611	0.5000	0.5987	0.7222	0.5000	0.5000	0.8645	0.9277	0.6325
GMM	0.6601	0.5483	0.5335	0.6917	0.7852	0.3933	0.6500	0.4349	0.4707	0.5975
DBSCAN	0.6976	0.000	0.5000	0.5000	0.5000	0.5000	0.5000	0.5000	0.5000	0.4108
LOF	0.5285	0.7704	0.4024	**0.8195**	0.8047	0.3933	0.6500	0.4954	0.5344	0.5998
Improvement (%)	18.31	2.19	6.57	-3.06	10.01	7.28	0.00	0.70	3.27	9.50
