# Supplementary material for: A Mallows-like criterion for anomaly detection with random forest implementation
Source: PLoS One. 2025 Jun 6;20(6):e0323333. doi: 10.1371/journal.pone.0323333 (PMC12143530; doi:10.1371/journal.pone.0323333)
Supplement: S3 Table — (PDF) [file pone.0323333.s003.pdf]

Table 3. Recall scores of anomaly detection algorithms

Model \ Dataset	SB	Pis	MHR	PS	PCO	Ye	Ca	MF	Sa	Mean
Modified Focal	**0.7606**	0.5993	0.1688	0.6211	**0.8025**	**0.1333**	**0.5000**	**0.9321**	**0.9106**	**0.6031**
Focal	0.4470	0.1569	0.0000	0.4203	0.6296	0.0000	0.3000	0.9179	0.8269	0.4110
Vote	0.4246	0.1417	0.0000	0.4119	0.6247	0.0000	0.2333	0.9121	0.8221	0.3967
Zero One	0.4470	0.1569	0.0000	0.4203	0.6296	0.0000	0.3000	0.9179	0.8269	0.4110
Hamming	0.4470	0.1569	0.0000	0.4203	0.6296	0.0000	0.3000	0.9179	0.8269	0.4110
Hinge Loss	0.4860	0.2569	0.0813	0.4439	0.6142	0.1083	**0.5000**	0.9038	0.8078	0.4669
Cross Entropy	0.4470	0.1569	0.0000	0.4203	0.6296	0.0000	0.3000	0.9179	0.8269	0.4110
Average	0.4470	0.1569	0.0000	0.4203	0.6296	0.0000	0.3000	0.9179	0.8269	0.4110
IF	0.0227	0.1250	0.2500	0.7783	0.7407	0.0000	**0.5000**	0.0000	0.0120	0.2699
Logistic	0.3863	0.0000	**0.5000**	0.0000	0.0000	0.0000	0.0000	0.0000	0.0000	0.0985
KNN	0.0455	0.1250	0.0000	0.2000	0.4444	0.0000	0.0000	0.7308	0.8554	0.2668
GMM	0.4545	0.2917	0.2500	0.5600	0.7407	0.0000	0.5000	0.0769	0.1446	0.3361
DBSCAN	0.6818	0.0000	0.0000	0.0000	0.0000	0.0000	0.0000	0.0000	0.0000	0.0757
LOF	0.2045	**0.7083**	0.0000	**0.8000**	0.7778	0.0000	0.5000	0.1923	0.2651	0.3609
Improvement (%)	11.60	-15.41	-66.24	-22.40	3.18	23.08	0.00	1.55	6.45	29.17
