# Supplementary material for: A Mallows-like criterion for anomaly detection with random forest implementation
Source: PLoS One. 2025 Jun 6;20(6):e0323333. doi: 10.1371/journal.pone.0323333 (PMC12143530; doi:10.1371/journal.pone.0323333)
Supplement: S4 Table — (PDF) [file pone.0323333.s004.pdf]

Table 4. F1-scores of anomaly detection algorithms

Model \ Dataset	SB	Pis	MHR	PS	PCO	Ye	Ca	MF	Sa	Mean
Modified Focal	**0.8522**	0.7356	0.2863	0.7500	**0.8812**	**0.2335**	**0.6562**	**0.9630**	**0.9490**	**0.6886**
Focal	0.6123	0.2691	0.0000	0.5834	0.7664	0.0000	0.4544	0.9554	0.9014	0.4876
Vote	0.5908	0.2464	0.0000	0.5753	0.7628	0.0000	0.3730	0.9522	0.8984	0.4847
Zero One	0.6123	0.2691	0.0000	0.5834	0.7664	0.0000	0.4544	0.9554	0.9041	0.4876
Hamming	0.6123	0.2691	0.0000	0.5834	0.7664	0.0000	0.4544	0.9554	0.9041	0.4876
Hinge Loss	0.6453	0.4034	0.1497	0.6045	0.7528	0.1944	**0.6562**	0.9456	0.8889	0.5587
Cross Entropy	0.6117	0.2691	0.0000	0.5828	0.7652	0.0000	0.4565	0.9537	0.9007	0.4875
Average	0.6123	0.2691	0.0000	0.5834	0.7664	0.0000	0.4544	0.9554	0.9014	0.4876
IF	0.0443	0.2184	0.3613	0.7317	0.7908	0.0000	0.5926	0.0000	0.0236	0.3187
Logistic	0.5519	0.0000	**0.5000**	0.0000	0.0000	0.0000	0.0000	0.0000	0.0000	0.1225
KNN	0.0868	0.2207	0.0000	0.3304	0.6097	0.0000	0.0000	0.8394	0.9187	0.3144
GMM	0.5940	0.6420	0.3920	0.6620	0.7800	0.0000	0.6250	0.1440	0.2530	0.4547
DBSCAN	0.6960	0.6520	0.0000	0.0000	0.0000	0.0000	0.0000	0.0000	0.0000	0.1508
LOF	0.3182	**0.7960**	0.0000	**0.8170**	0.8020	0.0000	0.6250	0.3240	0.4000	0.4876
Improvement (%)	22.44	-7.59	-42.74	-8.20	21.14	20.10	0.00	1.55	6.45	23.25
