# Supplementary material for: A Mallows-like criterion for anomaly detection with random forest implementation
Source: PLoS One. 2025 Jun 6;20(6):e0323333. doi: 10.1371/journal.pone.0323333 (PMC12143530; doi:10.1371/journal.pone.0323333)
Supplement: S5 Table — (PDF) [file pone.0323333.s005.pdf]

Table 5. Experimental results after data augmentation using ADASYN

Model \ Dataset	Original			Augmented		
	recall	AUC	F1-score	recall	AUC	F1-score
Modified Focal	**0.7606**	**0.8702**	**0.8522**	**0.8811**	**0.9056**	**0.9034**
Focal	0.4470	0.7231	0.6123	0.6947	0.8395	0.8256
Vote	0.4246	0.7121	0.5908	0.6845	0.8354	0.8264
Zero One	0.4470	0.7231	0.6123	0.6947	0.8395	0.8256
Hamming	0.4470	0.7231	0.6123	0.6947	0.8395	0.8256
Hinge Loss	0.4860	0.7355	0.6453	0.6799	0.8259	0.8134
Cross Entropy	0.4470	0.7355	0.6117	0.6947	0.8356	0.8234
Average	0.4470	0.7231	0.6123	0.6947	0.8395	0.8256
IF	0.0227	0.5071	0.0443	0.0000	0.4934	0.0000
Logistic	0.3863	0.6914	0.5519	0.8636	0.8905	0.8714
KNN	0.0455	0.5185	0.0868	0.5000	0.6996	0.6375
GMM	0.6601	0.4545	0.5940	0.2955	0.5764	0.4324
DBSCAN	0.6976	0.6818	0.6960	0.6818	0.6976	0.6960
LOF	0.5285	0.2045	0.3182	0.3409	0.5739	0.4824
Improvement (%)	9.03	25.87	22.44	2.03	1.70	3.67
