# Supplementary figures and images for: A Mallows-like criterion for anomaly detection with random forest implementation
Source: PLoS One. 2025 Jun 6;20(6):e0323333. doi: 10.1371/journal.pone.0323333 (PMC12143530; doi:10.1371/journal.pone.0323333)

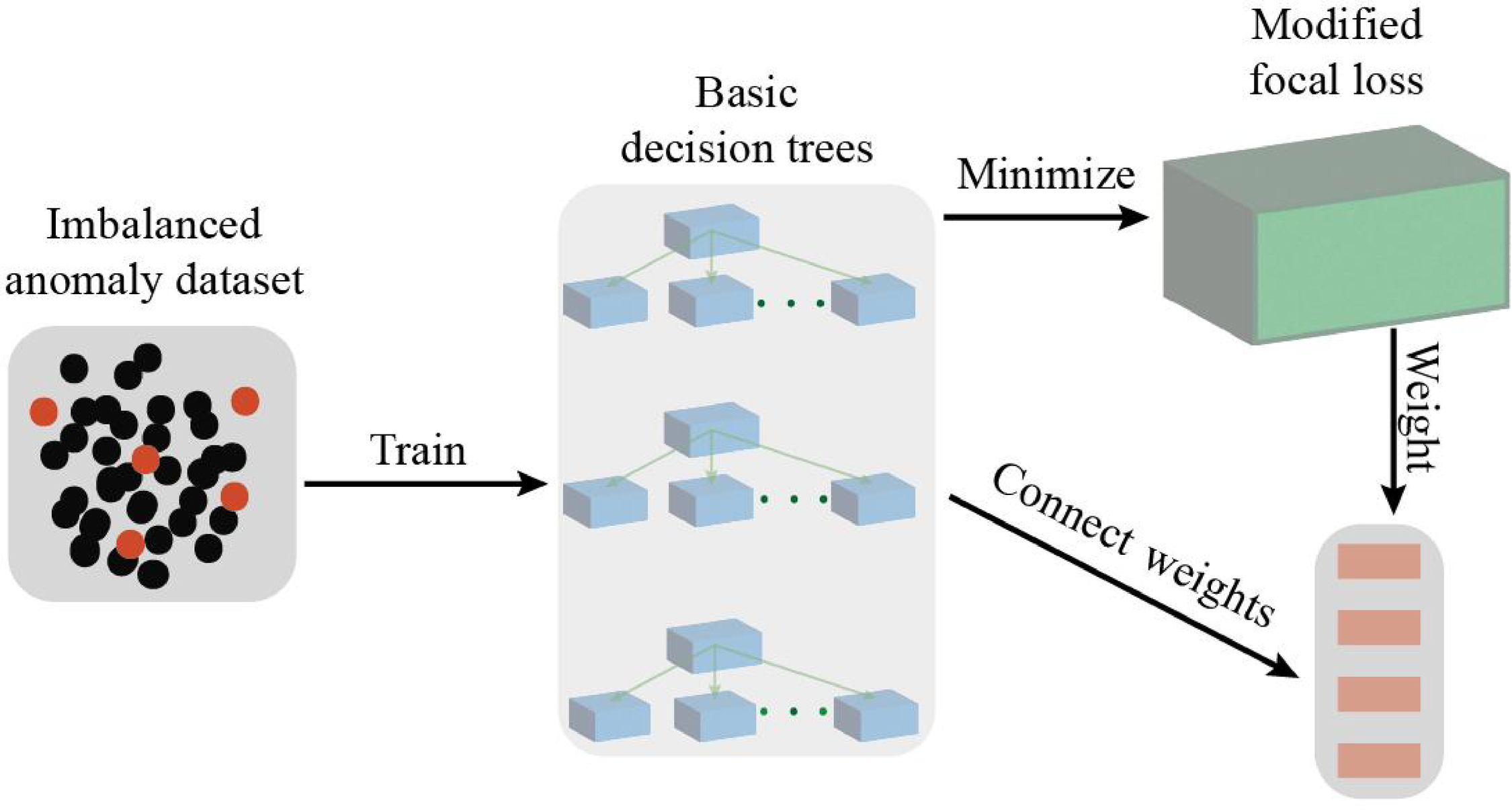

Supplement: S1 Fig — (TIF) [file pone.0323333.s006.tif]

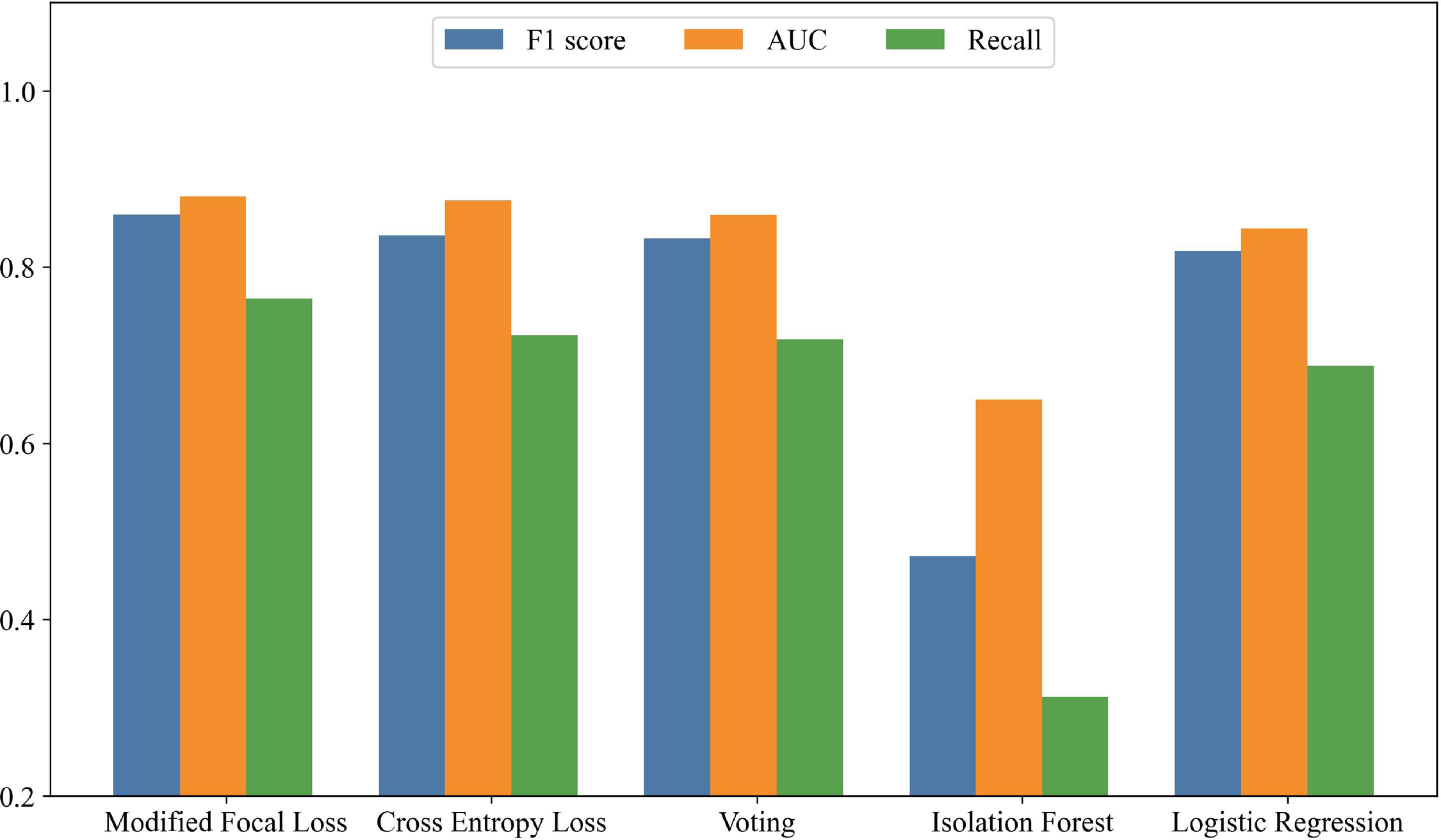

Supplement: S2 Fig — (TIF) [file pone.0323333.s007.tif]

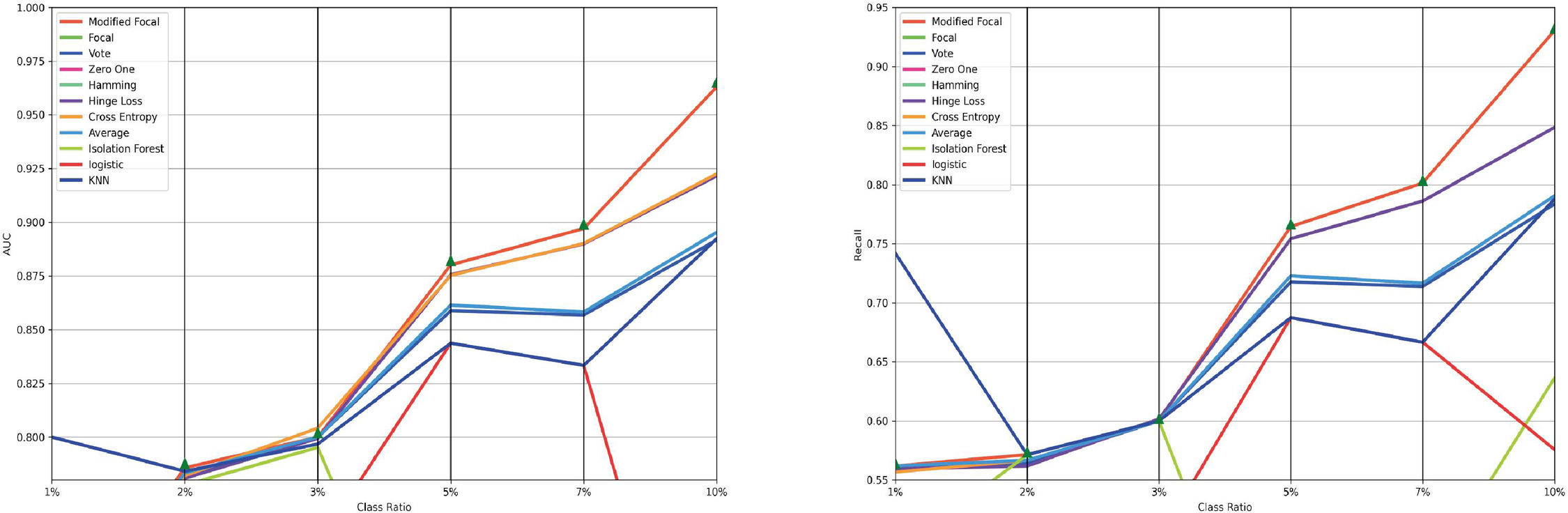

Supplement: S3 Fig — (TIF) [file pone.0323333.s008.tif]
