# Supplementary material for: A Mallows-like criterion for anomaly detection with random forest implementation
Source: PLoS One. 2025 Jun 6;20(6):e0323333. doi: 10.1371/journal.pone.0323333 (PMC12143530; doi:10.1371/journal.pone.0323333)
Supplement: Attachment — Submitted filename: Response to Reviewers.pdf [file pone.0323333.s009.pdf]

Responses to Reviewers' Comments for Manuscript PONE-D-24-52217

A Mallows-like Criterion for Anomaly Detection with Random Forest Implementation

Addressed Comments for Publication to

PLOS ONE

by

Gaoxiang Zhao, Lu Wang, Xiaoqiang Wang*

Response to Reviewer 1

Comment 1.1

The researchers mainly rely on Focal loss to address class imbalance, but I suggest testing other approaches (e.g., cost-sensitive learning or ensemble techniques such as ADASYN) to prove the quality of the method used, as using Focal loss may lead to certain drawbacks if the synthetic data points do not represent the actual minority class distribution.

Response 1.1: Done.

We are very grateful for your comment, which have inspired us to use the ADASYN technology to further verify the effectiveness of our algorithm. Please see more details in Section 3.2. In addition, we add three typical outlier detection method, that is LOF, DBSCAN, and GMM to compare with our method (see Table 2-4). All the results show that our method has advantages.

On the other hand, we have noticed that there are some differences between the method you mentioned and Ensemble learning method [1]. ADASYN, is one that approaches anomaly detection from the perspective of data. Nevertheless, ensemble learning methods enhance model performance through the integration of different base models. Data augmentation techniques like ADASYN can support all anomaly detection methods and are highly versatile, while ensemble methods focus more on the combination of base models. The advantages of ensemble methods can be found on Page 2, Lines 13-16.

Comment 1.2

The paper does not adequately mention how the features were selected, as the performance of machine learning models depends heavily on the quality of the input features.

Response 1.2: Done.

We have now addressed this by supplementing the details of feature selection of input datasets in Page 6, Lines 166-169. In addition, the process of generating subtrees in random forests already includes a feature selection step. Our model primarily focuses on model averaging of the random forest subtrees rather than the construction of the subtrees themselves. Therefore, we did not incorporate this feature selection step into our approach.

We appreciate your valuable suggestions and will incorporate feature selection into the model in the future, aiming for improved model performance.

Comment 1.3

Why did the researchers use mallow-like criterion in particular? Are there specific characteristics of the dataset that make this more suitable than others?

Response 1.3: Done.

The Mallows method is a typical approach in the field of model averaging. By minimizing the Mallows criterion, it maintains asymptotic optimality in the ensemble of linear or generalized linear regression problems. This method has been extended to certain machine

learning models such as random forests, where it also preserves asymptotic optimality. The detail description can be found in Page 2, Lines 36-46. In this work, we endeavor to extend the application of this methodology to classification problems.

Regarding your second concern, Our experiments on multiple datasets demonstrate that our method consistently outperforms in most scenarios. However, this characteristic merits further investigation to fully understand its implications and robustness.

Comment 1.4

Authors are advised to be precise in the abstract, and structure your abstract as follows-1) Background 2) Aim/Objective 3) Methodology 4) Results 5) Conclusion. Write 2-4 lines for each and merge everything in one paragraph (200-300 Words) without any subheading.

Response 1.4: Done.

Please check our revised abstract. Thank you very much.

Comment 1.5

The results were based on AUC, ARI, accuracy and recall. However, there are other important metrics for imbalanced datasets, such as F1 score and precision, which are very important in situation where false negatives are costly.

Response 1.5: Done.

Thank you very much for your valuable suggestions, we have replaced all evaluation metrics with the F1 score, AUC, and recall. Please chech the result in Page 6, Figure 2 and Page 7, Tabel 4. We observe that our algorithm shows a significant advantage on most datasets.

Comment 1.6

Authors should explain why the choice of Hierarchical sampling over other methods.

Response 1.6: Done.

We are very sorry for this description of sampling method, as we actually used simple random sampling instead of Hierarchical sampling. We have revised the problem in Page 6, Line 169. Thank you very much for your careful work.

Comment 1.7

Authors should state the hyperparameters and values of Bayesian model used in the research for hyperparameter tuning.

Response 1.7: Done.

We have addressed this by providing the specific hyperparameter for each datasets in Page 6, Table 1. Thanks for you suggestions.

Comment 1.8

Authors should create a new section in the experimental analysis section and compared the findings of the proposed model with existing works in the literature.

Response 1.8: Done.

We have addressed this by comparing our method against three additional techniques: LOF, DBSCAN, and GMM. Due to the presentation of the experimental section, we have incorporated these results into new rows within the original tables in Page 7, Table 2, Table 3 and Tabel 4. Thanks for your suggestion.

Comment 1.9

Incorporating relevant and recent academic sources could strengthen your paper's validity and give readers more context and background. Please avoid citing sources that were published before 2019. Cite current research that are really pertinent to your topic. The study also lacks sufficient citations. Authors can use and depend on these essential works while addressing the topic of their paper and current issues. Some latest papers listed below which studied similar problems can be added: 1). XIDINTFL-VAE: XGBoost-based intrusion detection of imbalance network traffic via class-wise focal loss variational autoencoder. 2). Feature selection in intrusion detection systems: a new hybrid fusion of Bat algorithm and Residue Number System. 3). Towards an efficient model for network intrusion detection system (IDS): systematic literature review. 4). GA-mADAM-IIoT: A new lightweight threats detection in the industrial IoT via genetic algorithm with attention mechanism and LSTM on multivariate time series sensor data.

Response 1.9: Done.

We have removed the research works from the manuscript that were not directly relevant, and we have cited several of the cutting-edge studies you provided, which we also consider pertinent. We have also incorporated a number of other cutting-edge research articles. Given that our method is framed within a model averaging approach, some referenced works may be relatively earlier contributions to the field. We have made our effort to correct and refine these aspects within the manuscript. Thanks for your suggestion.

Comment 1.10

In the end of related work section, highlight in 10-15 lines what overall technical gaps are observed in existing techniques that led to the design of the proposed methodology.

Response 1.10: Done.

In the introduction section, we have added a discussion on the limitations of previous work in Page 1, Lines 9-12 and Page 2, Lines 36-46, where we discuss the limitations of ensemble methods and the Mallows criterion in the field of anomaly detection separately.

Comment 1.11

Although the English is generally quite good, there are quite a few minor grammatical errors, and a careful read-through is needed to eliminate these errors. The spelling mistake should be corrected by reading through the manuscript.

Response 1.11: Done.

We have thoroughly proofread and revised the language content of the manuscript. In addition, we also invited native English speakers to help us revise the paper(Lethub unique code : PRL_250210H532F).

Comment 1.12

Please Change the “conclusion” section to “Conclusion and Future Work” and write future work.

Response 1.12: Done.

References

[1] Charu C. Aggarwal. *An Introduction to Outlier Analysis*, pages 1–34. Springer International Publishing, Cham, 2017.
